# Combining qualitative research with PPI: reflections on using the person-based approach for developing behavioural interventions

**DOI:** 10.1186/s40900-019-0169-8

**Published:** 2019-11-14

**Authors:** Ingrid Muller, Miriam Santer, Leanne Morrison, Kate Morton, Amanda Roberts, Cathy Rice, Marney Williams, Lucy Yardley

**Affiliations:** 1 Primary Care and Population Sciences, University of Southampton, Southampton, UK; 2 Academic Unit of Psychology, University of Southampton, Southampton, UK; 3 Patient and Public Contributor, Southampton, UK; 4 School of Experimental Psychology, University of Bristol, Bristol, UK

**Keywords:** Person-based approach, Patient and public involvement, Qualitative research

## Abstract

**Background:**

The value and importance of qualitative research and Patient and Public Involvement (PPI) for developing complex health interventions is widely recognised. However, there is often confusion between the two, with researchers relying on just one of these approaches, rather than using the two alongside one another.

**Methods:**

The Person-Based Approach (PBA) to developing health-related behaviour change interventions adapts and integrates methods from user-centred design and qualitative research. The PBA involves qualitative research at multiple stages of interventions to ensure they are acceptable, feasible, meaningful, and optimally engaging to the people who will use them. The qualitative research is carried out with research participants from a target population, who have no prior or continuing involvement in the wider research process and see the intervention from a fresh perspective. This enables in-depth understanding of the views and experiences of a wide range of target users and the contexts within which they engage with behavioural change.

PPI in research is carried out *with* or *by* members of the public and is a key part of the research process. PPI contributors are involved at all stages of research design and interpretation. PPI provides input into interventions as members of the research team alongside other stakeholders, such as health professionals and behaviour change experts.

**Results:**

We advocate using qualitative research alongside PPI at all stages of intervention planning, development, and evaluation. We illustrate this with examples from recent projects developing complex health interventions, highlighting examples where PPI and PBA have pulled in different directions and how we have approached this, how PPI have helped optimise interventions based on PBA feedback, and how we have engaged PPI in community settings.

**Conclusions:**

PPI provides a valuable alternative to the traditional researcher-led approaches, which can be poorly matched to the needs of target users. Combining PPI with the PBA can help to create optimally engaging interventions by incorporating a greater diversity of feedback than would have been possible to achieve through PPI or qualitative approaches alone.

## Plain English summary

Qualitative research methods and Patient and Public Involvement (PPI) are widely recognised as important for developing interventions aimed at improving health. However, there is often confusion between the two, with research teams favouring one approach over the other. Terms like “getting user feedback” and “focus groups” are often used to describe both PPI and qualitative research methods, adding to confusion between the two approaches.

The Person-Based Approach (PBA) to developing interventions uses qualitative research at every stage of developing and testing interventions to ensure they are meaningful, useable, and engaging to the people who will use them. People who take part in qualitative interviews are research participants and are not involved in research processes, such as deciding how to incorporate feedback.

PPI contributors are members of the research team, involved in all research processes such as applying for research funding, planning and designing the intervention, giving feedback on draft materials, and interpreting research findings. Qualitative participants and PPI contributors have different and complementary roles, and we advocate using qualitative research alongside PPI at all stages of developing and testing health interventions.

This paper illustrates our approach through recent examples of developing health interventions, highlighting examples where PPI and PBA have pulled in different directions and how we have approached this. Using the PBA alongside PPI can lead to more engaging interventions by including a greater diversity of feedback than would have been possible to achieve through PPI or qualitative research approaches alone.

## Background

There has been increasing recognition of the value of both qualitative methods and Patient and Public Involvement (PPI) in the development of complex behavioural interventions [1–3]. Qualitative methods can provide vital insight into the design of an intervention, ensuring it is acceptable and engaging to users and embedded in the context in which it will be used [2, 4, 5]. Qualitative and mixed-methods research in intervention development ranges from innovative methods of synthesising research evidence, such as critical interpretative synthesis [6] and realist reviews [7], to applied or theory-driven primary qualitative work. Qualitative research methods enable researchers to explore the views and perspectives of a wide range of people within a target group, helping identify the key behavioural issues, needs, and challenges.

The Person Based Approach (PBA) to intervention development [4] uses qualitative research at various stages to develop health-related behaviour change interventions. It is an iterative approach which emerged over the past decade, and continues to evolve as we find more effective ways of implementing it. This approach to intervention development has been used alongside PPI to create numerous engaging and effective behavioural interventions for a wide range of populations and health conditions [8–10]. See website www.personbasedapproach.org for more information. A major strength of this approach is the way it enables input and comments from a wide range of target users at multiple stages of intervention development to ensure all intervention components are acceptable, feasible, meaningful, and optimally engaging.

PPI is defined as research being carried out *with* or *by* members of the public rather than *to*, *about*, or *for* them [3]. PPI contributors may be patients, members of the public, carers, people who use health and social care services, or members of organisations representing service users. The contributions of members of the public and patients are crucial in applied health research as they offer a valuable alternative to the opinions and views of researchers and healthcare professionals, which can improve the design and conduct of research. PPI also has important ethical value in making sure that the views of patients and service users are represented in research, and helping ensure research is focussed on aspects that are important to patients. In intervention development, PPI contributors are often members of the research team involved in all aspects of the project, from prioritising research topics and securing funding to interpreting and disseminating research findings. PPI contributors may help decide what interventions are needed, input on which intervention components might be useful and how they could be applied, and help interpret qualitative data during intervention development and optimisation. Research teams often rely on Patient and Public Involvement (PPI) to provide insight into the context within which users are likely to engage in behaviour change, and improve the quality and acceptability of interventions.

There is an emerging body of evidence on the impact of PPI in health and social care research, however this evidence is currently limited due to issues surrounding the measurement, evaluation, and reporting of PPI impact [11–13]. Systematic reviews of the literature have found that PPI enhances the quality and appropriateness of research at all stages, from planning and conceptualisation through to implementation and dissemination [14]. PPI in research has also been found to improve chances of securing research funding, increase participant recruitment rates, aid protocol development, and help choose acceptable and appropriate outcome measures [11].

There is considerable confusion between the use of qualitative research methods in intervention development and PPI contribution. For example, terms such as ‘focus groups’ or ‘eliciting user feedback’ are commonly used for both qualitative research and for eliciting views in PPI. Co-design, co-creation [15] or participatory research [16], are methods of PPI that broadly aim to engage stakeholders, including potential users of the proposed intervention to work in partnership. These processes may be more adaptive and rapid than applying qualitative research (where the people involved are research participants rather than PPI contributors) to intervention development.

While the terminology around qualitative methods, PPI contribution and co-design sometimes overlap, there can also be an overlapping and lack of clarity about roles. A recent review of PPI in research found that research participants are often asked to also act as PPI on the project. These dual roles can be blurred, which can cause confusion about whether the person is speaking as a patient, participant, advisor, or member of the research team [17]. PPI does not require research ethics approvals, which is thought to occasionally lead to unintentionally unethical practices [18]. Differentiating qualitative research from PPI has been identified as a core way of overcoming potential ethical issues [18], highlighting the importance of considering how to utilise these approaches most effectively when developing behavioural interventions.

This paper will consider the important contributions of both qualitative research and PPI to developing complex health interventions, reflecting on our experience of using the PBA [4] alongside working with PPI contributors when developing interventions. The paper focuses on a pragmatic approach to working with PPI in order to optimise research design, conduct and outcomes, but also touches on some of the ethical aspects of PPI.

## Methods

There is an increasing movement towards using PPI or co-design approaches (in which PPI and researchers design the intervention together) [15, 16] instead of qualitative research methods. Advocates of co-design design have promoted this approach as a way of engaging lay communities in research while meeting the aim of producing research that is acceptable and meets the needs of users. There may be a risk that this approach to developing complex interventions can lead to an over-reliance on ‘PPI preference’ as justification for including aspects of an intervention, rather than including theory and evidence to inform intervention development in addition to PPI.

**Fig. 1:**
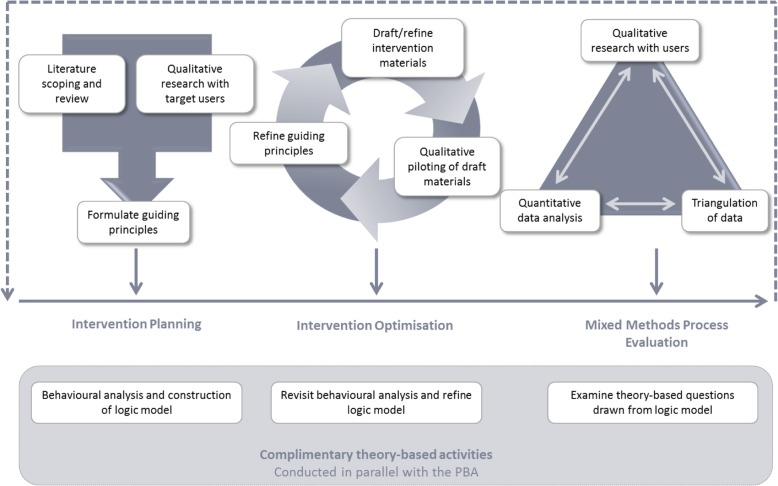
Overview of the Person-Based Approach to intervention development

The PBA to intervention development adapts and integrates methods from user-centred design, qualitative, and mixed-methods research. This approach to developing interventions enables an in-depth understanding of the views and experiences of intervention users and the contexts within which they engage with behavioural change. The PBA applies a systematic approach to intervention development, as per best practice guidelines [19], and can be applied to all stages of developing complex interventions. The PBA is an iterative process to be used alongside behavioural theory and analysis. The process can be divided into three stages: intervention planning, intervention optimisation, and process evaluation. See Fig. 1 for an overview of the PBA.

During the intervention planning stage, qualitative methods are used to inform the intervention design to ensure it is optimally engaging and persuasive. If appropriate, published qualitative research can be used to extract key barriers and facilitators to behaviour change, and to provide insight into the contexts in which people may engage with the intervention. Primary qualitative research may be needed if the existing literature is insufficient. Insights gained from this qualitative work are then used to formulate guiding principles for developing the intervention. The guiding principles specify ‘*what*’ the design objectives must be, and ‘*how*’ they may be achieved. They are grounded in our understanding of the people who will use the intervention and the contexts within which they are likely to engage in the behavioural change. Once formulated, the guiding principles offer a clear summary of the ways in which the intervention will maximise engagement and support behaviour change [4].

Qualitative or mixed-methods research is also used during intervention optimisation to elicit target users’ views and experiences of using the draft intervention. An inductive approach to qualitative research is taken where the researcher is guided by the emerging data, rather than pre-existing assumptions. This helps understand the ways in which people engage with the materials and key messages. We often use qualitative think-aloud interviews during intervention optimisation. This qualitative technique involves people from a target group using the intervention materials as they normally would while saying all their thoughts out loud. This qualitative method provides insight that goes beyond assessing the usability and acceptability of interventions. Crucially, think aloud interviews can highlight issues around participant engagement with behavioural advice and the behaviour change process facilitated by the intervention materials [20, 21].

The PBA can also be applied to the process evaluation of complex interventions, using qualitative methods to understand participants’ experiences of using a fully deployed intervention and engaging in behaviour change [22]. This qualitative research is often triangulated with quantitative intervention data to create a greater understanding of how and why people engaged with the intervention in a certain way, helping guide implementation of an intervention or further modification if needed.

The PBA may appear resource intensive, but in our experience investing time identifying and resolving issues around user engagement avoids developing and evaluating interventions that will not be engaging or effective. The PBA is intended to be applied flexibly to intervention development, depending on the methods and resources available. For example, the existing literature may be reviewed as a rapid scoping review and qualitative interviews in the intervention planning and process evaluation phases may be carried out via the telephone if needed. Think aloud interviews offer crucial insight into how users engage with interventions, but, depending on the size and scope of the intervention, interviewing as few as 5–10 people may provide sufficient data for optimisation. Furthermore, components of the PBA could be carried out as part of student research projects if appropriate.

Throughout the PBA, the research is carried out with research participants. Participants’ opinions, views, and experiences are immensely valuable, in part because these research participants have not been involved in the research process and therefore are viewing intervention materials from a ‘fresh’ or neutral perspective. For example, research participants have not been involved in decisions about design or about how best to incorporate feedback into interventions, data analysis, or dissemination. PPI members of the research team are likely to have been involved in the study over a long period of time, helping make intervention development decisions and will be completely familiar with the aims of the interventions. PPI contributors are often highly invested in the research, and so may be less able to view intervention materials critically compared to research participants.

We advocate conducting qualitative research alongside PPI at all stages of intervention development. The following section will illustrate how our research team has done this successfully in previous projects.

## Results

We have developed complex behavioural interventions using the PBA for a wide range of populations, conditions, and contexts. In addition to using the PBA to intervention development, we also work closely with PPI contributors within the research team. We benefit from their input at all stages of research, from topic prioritisation and securing funding, to data analysis and dissemination. During intervention development, PPI contributors are invited to provide input on all aspects of the protocol, interview guides and other study materials, as well as early iterative input into intervention materials. When commenting on draft intervention materials we tabulate all feedback from PPI and other expert team members (e.g. clinicians, health researchers) for consideration alongside other sources such as theory, evidence, and qualitative participant data. The research team then discusses and reaches a consensus about which feedback to incorporate and how best to do this. This section provides three illustrations of how we have combined PPI feedback with the PBA when developing complex interventions.

**Illustration 1:** Combining PPI and PBA feedback and resolving tensions where they arise

Eczema Care Online (ECO) is a NIHR funded programme (PR-PG-0217-20,007) involving the development and evaluation of two online interventions to support eczema self-care: one for parents / carers of children with eczema aged 0–12 years, and one for young people aged 13–25 years. The interventions were developed following the Person-Based Approach (PBA) to intervention development, alongside PPI input.	
The ECO project team includes two adult PPI contributors who have been involved from the very start of planning the funding application. Once funding was secured, we also involved two youth PPI contributors in the project. Not all PPI contributors were able or wished to be involved in all aspects of the project. For example, our youth PPI contributors were only involved in some of the tasks below, but their input was still invaluable throughout intervention development.	
Our two adult PPI colleagues are fully integrated into the research team. They are invited to all management meetings and are included on all project circulations. Our PPI contributors are invited to input on all aspects of the project, including all protocol discussions and co-authoring outputs. As part of our intervention development group, which also includes clinicians, psychologists, and skin researchers, our PPI contributors have been involved in:	
• Developing intervention objectives	
• Defining target user characteristics and intervention guiding principles	
• Developing qualitative interview guides	
• Nomenclature of eczema treatments such as topical corticosteroids (flare control creams)	
• Interpreting key messages from qualitative research and systematic reviews	
• Intervention planning and design	
• Commenting on all draft intervention materials	
• Planning the trial of the interventions	
• Planning process evaluation	
Development of the ECO interventions followed the PBA. We conducted a systematic review of the qualitative literature, a secondary analysis of qualitative interviews with young people with eczema, and primary qualitative interviews with parents of children (aged 0–12 years) with eczema, children (aged 6–12 years) with eczema, and young people (aged 13–25 years) with eczema. This research enabled us to develop our intervention guiding principles. It also informed the theory-based activities we carried out alongside the PBA, such as identifying barriers and facilitators to target behaviours as part of our behavioural analysis, and constructing a logic model of how we anticipate the intervention to result in behaviour change and core trial outcomes.	
During intervention optimisation, draft intervention materials were assessed in qualitative think aloud interviews. We selected participants to ensure we included a wide range of people. The intervention was optimised through an iterative process, incorporating user feedback where possible. Data from qualitative participants was considered alongside feedback from the intervention development group, which included PPI, clinicians, Health Psychologists, and skin researchers.	
The intervention development group provided feedback that was incorporated into the draft materials, although tensions between their feedback and data from qualitative participants arose at times. For example, our intervention development group favoured the use of medical terminology and giving participants all the information they may need. Participants in the think aloud interviews, however, sometimes found medical terminology off-putting and often found the volume of information overwhelming.	
A core aspect of the interventions was a series of videos developed to reinforce target behaviours. We worked closely with our PPI contributors to develop the initial video scripts and storyboards. We also received additional PPI input on the video development through the Nottingham Centre of Evidence Based Dermatology’s patient panel, alongside input from research participants in qualitative think aloud interviews. While research participants and PPI feedback concurred on many points and both shaped the development of the videos, the feedback differed in some respects. For instance, members of the patient panel thought that the messages in the videos were too basic and perhaps a little childish. However, participants in the think aloud interviews felt that the videos were one of the most helpful aspects of the intervention with many saying the content was novel and that it helped explain key messages. Members of the patient panel are often particularly well informed about their condition and in this instance; we felt that the feedback from the research participants might be a stronger indication of how the videos would be received by the target audience of people fairly new to managing eczema.	
Views emerging from our research participants were usually given precedence over the views of our intervention development group, unless it was deemed medically inaccurate or potentially harmful. When these tensions arose, we had open and frank discussions with our PPI contributors and other colleagues in the intervention development group who were fully supportive of following the PBA to ensure the interventions are grounded in the views and perspectives of the people who will use them.

**Illustration 2:** Working with PPI contributors to optimise the intervention based on PBA user feedback

STREAM (Screen and TREAt for Malnutrition) is a NIHR funded programme (RP-PG-0614-20,004) involving the development and evaluation of interventions to support screening and treatment of malnutrition risk within primary care, among older adults living in the community. Two interventions were developed: an online training tool for Health Care Professionals and a booklet-based intervention for older adults (aged over 65 years) to motivate and support change in eating patterns. The interventions were developed following the Person-Based Approach (PBA) to intervention development, alongside PPI input.	
The STREAM project team included three PPI contributors, two of whom were involved from early stages of funding application with the third joining after funding was secured. All PPI contributors are fully integrated within the research team and are invited to attend all management meetings and input on all aspects of the intervention, alongside other members of the intervention development group including clinicians, psychologists, nutritionists, dieticians, nurses, and gerontologists. Our PPI contributors have been involved in various tasks, including:	
• Developing intervention objectives	
• Defining target user characteristics and intervention guiding principles	
• Developing qualitative interview guides, participant information sheets, and consent forms	
• Interpreting key messages from qualitative research and systematic reviews	
• Intervention planning and design	
• Commenting on all draft intervention materials	
• Planning the feasibility trial of the intervention	
Older adults’ reactions to and experiences of using the draft booklets were explored using qualitative think aloud interviews. The design and content of the booklets were optimised through an iterative process, with later participants viewing booklets that were revised based on earlier participants’ feedback. Data from qualitative participants were discussed with and considered alongside feedback from our intervention development team.	
Early contributions from our PPI were crucial for enabling us to efficiently develop and refine first drafts of the booklets that provided nutritional advice more relevant to the experiences of older adults. In particular, our PPI contributors provided advice on enhancing the relevance of eating well in older adulthood (e.g. maintaining energy, strength and independence) and improving the communication of key messages (e.g. language, complementary imagery, branding).	
Our qualitative research enabled us to seek further feedback from a wider range of target users who had additional, varied experiences contributing to their appetite and eating patterns (e.g. recent bereavements, health conditions, hospital stays etc.). It was clear from this qualitative research that the key messages within the booklet were received poorly by some participants. For example, advice on how much food and drink older adults needed to consume was perceived to be unrealistic and overwhelming, undermining any further engagement with the intervention. In discussion with our PPI contributors and intervention development team, we experimented with various language tweaks (e.g. rephrasing ‘meals’ as ‘bites’). After these tweaks, the key messages were received favourably by participants.	
For STREAM, PPI contribution was critical for facilitating early intervention planning and development, and considering how best to optimise booklets based on user feedback. However, without additional qualitative research, we likely would not have identified and addressed the potential for poor engagement with the key intervention messages ahead of a feasibility trial.

**Illustration 3:** Incorporating feedback from PPI within community settings to intervention development

*Aims*	
This example will focus on how PPI feedback from community settings was incorporated with insights from the PBA and PPI contributors on the research team during intervention development. The programme of research is funded by the Stroke Association and British Heart Foundation, and seeks to develop a digital intervention for patients and GPs to reduce raised blood pressure after a stroke or Transient Ischaemic Attack (TIA).	
*Methods/What we did*	
PPI contributors on the research team raised the importance of consulting with a diverse range of stroke patients (e.g. in terms of fatigue, and cognitive or mobility problems) when designing the intervention to promote feasibility and acceptability. Our PPI contributors helped put us in touch with community support groups, and the Different Strokes Southampton group and Oxford Aphasia Group kindly invited us. We visited each group three times during the early stages of intervention planning and development - speaking to more than 30 PPI contributors in community settings. Unlike our qualitative research using the PBA, these sessions were not recorded and did not seek to achieve detailed feedback on every aspect of the intervention. Instead, a flexible set of questions were prepared to facilitate an informal discussion about the intervention and target behaviours, at times supported by extracts from the intervention or recruitment materials which we were particularly interested in exploring with our target population.	
*Results/What we discovered*	
PPI contribution from these community groups was extremely valuable to help inform decisions about the intervention design and procedures. For example, while evidence had suggested that daily reminders to monitor blood pressure could be irritating, discussions with stroke patients and their carers suggested that daily reminders would be important, and some wanted the option for twice daily reminders. This suggested the need for a flexible reminder system.	
*Implications*	
Engaging with PPI in community settings provided additional insights to complement working with PPI within the research team, and using the PBA. We gained rapid feedback at an early point of intervention development from a diverse group of people, informing key decisions about the draft intervention. Discussing the specific nature of this intervention with a wide range of people with diverse experiences of stroke highlighted novel concerns which had not emerged from the evidence, or from our research planning meetings.	
Community group settings appeared most useful for generating ideas and raising concerns, rather than refining the intervention content. For this, the feedback from community groups was discussed with the intervention development team, and our PPI contributors within the team were essential in deciding how best to implement changes to the intervention to accommodate these novel perspectives. Qualitative research conducted in line with the PBA then enabled more in-depth exploration of perceptions of the intervention content, informing further optimisation of the intervention.	

The three studies described in these illustrations all used the PBA alongside PPI to develop complex behavioural interventions. While each illustration reflects on an aspect of the process that was particularly important to that study, the broad issues described are applicable to all the interventions we develop using the PBA. For example, the views and experiences of PPI contributors do not always match those of our other team members or qualitative research participants. We find it helpful to enter all feedback from research participants, PPI, health professionals, researchers, and other relevant sources into a table. This provides a systematic approach to documenting, categorising, and prioritising all suggested changes, and the table is useful for facilitating open discussions about changes to the interventions with PPI and other colleagues. See [23] for more detail about using tables to document and categorise feedback in the PBA.

## Discussion

There has been a drive to increase PPI in health research in order to improve prioritisation of research topics, enhance acceptability of research to participants, and maximise the quality and dissemination of research. In the development of complex interventions, PPI can also ensure an understanding of the context within which interventions may be used as well as an understanding of the needs of users. PPI provides a valuable alternative to researcher-led approaches where the agenda is set by research teams, which can be poorly matched to the needs, views, and values of target users.

However, PPI may sometimes be used as an alternative to qualitative research as a way of obtaining user feedback. This can risk crossing ethical boundaries [18] and can be problematic as PPI contributors are often members of the research team who are invested in the research project and have an understanding of the aims of the intervention, which may not be apparent to a user who is not familiar with the intervention. Furthermore, PPI is often provided by a small number of dedicated and articulate individuals who may not be typical of the whole target population, especially hard to reach and socially deprived groups, (although it is acknowledged that this social bias can also be present amongst respondents who participate in research). Efforts to increase diversity in PPI contributors are welcome, but even when involving large PPI panels, it may not be possible to ensure that the wide range of views, perspectives, and experiences that the research team needs to consider will be represented. Intervention developers need to engage with the widest range of views possible. This may involve conducting qualitative research alongside PPI to include those who do not have the time, skills and interest to become PPI contributors. In some populations, however, people may prefer to contribute to discussions without consenting to becoming research participants.

We advocate combining PPI with qualitative research. This enables engagement with a wide range of target users through purposive sampling and overcomes the ‘group think’ that can arise through familiarity and involvement in the research project and intervention development that may develop with PPI representatives or the risk that the ‘lay’ voices of PPI contributors become less ‘lay’ through increasing research experience [24]. Research participants in the qualitative work advocated by the PBA are typically new to the intervention, with no prior knowledge or understanding of the research aims or early draft intervention materials, offering a fresh perspective for evaluating iterative changes, and in-depth understanding of how people are engaging with the intervention.

## Conclusion

Qualitative research methods and PPI serve different, complementary functions for developing complex health interventions. Using the PBA alongside PPI can lead to optimally engaging interventions by incorporating a greater diversity of feedback than would have been possible to achieve through PPI or qualitative approaches alone.

## Data Availability

Data sharing is not applicable to this article as no datasets were generated or analysed during the current study.

## Abbreviations

ECO: Eczema Care Online
PBA: Person-Based Approach
PPI: Patient and Public Involvement
STREAM: Screen and Treat for Malnutrition
